# A phase Ib study of capecitabine and ziv-aflibercept followed by a phase II single-arm expansion cohort in chemotherapy refractory metastatic colorectal cancer

**DOI:** 10.1186/s12885-019-6234-8

**Published:** 2019-11-01

**Authors:** John H. Strickler, Christel N. Rushing, Donna Niedzwiecki, Abigail McLeod, Ivy Altomare, Hope E. Uronis, S. David Hsu, S. Yousuf Zafar, Michael A. Morse, David Z. Chang, James L. Wells, Kimberly L. Blackwell, P. Kelly Marcom, Christy Arrowood, Emily Bolch, Sherri Haley, Fatima A. Rangwala, Ace J. Hatch, Andrew B. Nixon, Herbert I. Hurwitz

**Affiliations:** 1 Duke University Medical Center, Box 3216, 30 Duke Medicine Circle, Room #0050, Durham, NC 27705 USA; 2 Virginia Oncology Associates, Hampton, VA 23666 USA; 3 Lexington Oncology Associates, West Columbia, SC 29169 USA; 4 Duke Cancer Institute Biostatistics and Bioinformatics, Duke University Medical Center, Durham, NC 27705 USA; 5 Shattuck Labs, Research Triangle Park, Durham, NC 27709 USA; 6 Genentech, Inc., South San Francisco, CA 94080 USA

**Keywords:** Capecitabine, Ziv-aflibercept, Metastatic colorectal cancer, Advanced solid tumors

## Abstract

**Background:**

Patients with chemotherapy refractory metastatic colorectal cancer (CRC) have a poor prognosis and limited therapeutic options. In this phase Ib/II clinical trial, we established the maximum tolerated dose (MTD) and recommended phase II dose (RPTD) for the combination of capecitabine and ziv-aflibercept, and then we evaluated the efficacy of the combination in patients with chemotherapy refractory metastatic CRC.

**Methods:**

All patients were required to have a Karnofsky Performance Status > 70% and adequate organ function. The phase Ib dose escalation cohort included patients with advanced solid tumors who had progressed on all standard therapies. Using a standard 3 + 3 design, we identified the MTD and RPTD for the combination. Fifty patients with metastatic CRC who had progressed on or were intolerant of a fluoropyrimidine, oxaliplatin, irinotecan, and bevacizumab were then enrolled in a single-arm phase II expansion cohort, and were treated at the RPTD. Prior EGFR antibody therapy was required for subjects with *RAS* wildtype tumors. The primary endpoint for the expansion cohort was progression-free survival (PFS) at two months. Secondary endpoints included objective response rate (ORR) and overall survival (OS).

**Results:**

A total of 63 patients were enrolled and evaluable for toxicity (13 dose escalation; 50 expansion). The MTD and RPTD were: capecitabine 850 mg/m2, P.O. bid, days 1–14, and ziv-aflibercept 6 mg/kg I.V., day 1, of each 21-day cycle. In the expansion cohort, 72% of patients were progression-free at two months (95% confidence interval [CI], 60–84%). Median PFS and OS were 3.9 months (95% CI, 2.3–4.5) and 7.1 months (95% CI: 5.8–10.0), respectively. Among all patients evaluable for toxicity, the most common treatment related adverse events (all grade [%]; grade ≥ 3 [%]) included palmar-plantar erythrodysesthesia (41%; 6%), hypertension (33%; 22%), and mucositis (19%; 5%). RNA was isolated from archived tumor specimens and gene expression analyses revealed no association between angiogenic biomarkers and clinical outcomes.

**Conclusion:**

The combination of capecitabine and ziv-aflibercept at the RPTD demonstrated acceptable safety and tolerability. PFS at 2 months in patients with chemotherapy refractory metastatic CRC was significantly greater than that in historical controls, indicating that this combination warrants further study.

**Trial registration:**

This clinical trial was registered in the www.clinicaltrials.gov system as NCT01661972 on July 31, 2012.

## Introduction

Colorectal cancer (CRC) is the fourth leading cause of cancer death in men and the third leading cause of cancer death in women worldwide [1]. Once a patient with metastatic CRC has experienced progression on first and second-line chemotherapy and biological therapies, the survival benefit of remaining therapies is limited [2, 3]. In addition, therapeutic options may be constrained by patient comorbidities, age, or performance status. Novel therapeutic regimens are needed that are both tolerable and provide meaningful clinical benefit.

Ziv-aflibercept is a recombinant fusion protein containing portions of the extracellular domains of VEGFR1 (also known as Flt-1) and VEGFR2 (also known as KDR or Flk-1). Ziv-aflibercept binds vascular endothelial growth factor (VEGF)-A, VEGF-B, and placental growth factor (PlGF), thereby inhibiting VEGF-mediated angiogenesis [4]. Ziv-aflibercept is approved by the United Stated Food and Drug Administration (FDA) and the European Medicines Agency (EMA) for use in combination with Fluorouracil (5-FU), leucovorin, and irinotecan (FOLFIRI) for the second line treatment of metastatic CRC [5]. In the phase III VELOUR trial, the addition of ziv-aflibercept to FOLFIRI in patients with metastatic CRC resulted in a survival of 13.5 months, compared to 12.1 months for FOLFIRI alone (hazard ratio [HR], 0.82; 95% confidence interval [CI], 0.71–0.94; *P* < 0.01) [6].

Capecitabine is an oral fluoropyrimidine carbamate indicated for the treatment of metastatic CRC [7]. Capecitabine is considered interchangeable with intravenous 5-FU based on its similar efficacy and safety [8–12]. In the chemotherapy refractory setting (3rd line and beyond), capecitabine monotherapy has a median time to progression of 2 months [13]. Prior studies combining capecitabine with the anti-VEGF monoclonal antibody bevacizumab have shown that the combination is clinically active and tolerable, even among patients with advanced age or comorbidities [14, 15]. Since anti-VEGF therapies are active for patients with metastatic CRC in the first-line [16], second-line [17, 18], and beyond [2], we hypothesized that the combination of capecitabine and ziv-aflibercept would be tolerable, and would exceed the PFS of historical controls.

In this phase Ib/II clinical trial, we assessed the safety, tolerability, and efficacy of capecitabine in combination with ziv-aflibercept. The primary objective was to establish a maximum tolerated dose (MTD) and a recommended phase two dose (RPTD) in patients with advanced solid tumors. We then enrolled 50 patients with chemotherapy refractory metastatic CRC in a single-arm phase II expansion cohort to establish the safety and efficacy of the RPTD.

## Patients and methods

### Study design

This multi-center phase Ib/II clinical trial was conducted at Duke University Medical Center (Durham, North Carolina), Duke Cancer Network clinical sites, Virginia Oncology Associates (Hampton, Virginia), and Lexington Medical Center (West Columbia, South Carolina). This study was performed after approval by the Institutional Review Boards of participating centers. All patients provided written informed consent prior to any study-related procedure. This study was conducted in accordance with guidelines of the Helsinki Declaration, and is registered with ClinicalTrials.gov (NCT01661972).

This study consisted of a Phase Ib (dose escalation) cohort followed by a Phase II expansion cohort. In the Phase Ib dose escalation cohort, we utilized a standard “3 + 3” design to identify the MTD and RPTD of the combination of capecitabine (Genentech, South San Francisco, CA, USA) and ziv-aflibercept (Sanofi-Aventis, Bridgewater, NJ, USA) in patients with advanced solid tumors. The MTD was defined around toxicity occuring in the first cycle. The RPTD was selected based on safety and tolerability in all cycles.

In the Phase II expansion cohort, we treated 50 subjects with chemotherapy refractory metastatic CRC to determine the safety, tolerability, and clinical activity of capecitabine in combination with ziv-aflibercept. The dose and schedule of each therapy was based on the RPTD from the Phase Ib dose escalation cohort, and are listed in Table 1. Treatment was continued for all patients until disease progression, unacceptable toxicity, or death.

**Table 1:** Dose and schedule of Capecitabine and Ziv-aflibercept

Cohort	# Evaluable Subjects	Capecitabine P.O. (mg/m^2^) BID, Days 1–14	Ziv-Aflibercept I.V. (mg/kg) Q3 weeks, Day 1
Phase Ib (dose escalation)			
Cohort 1	7	850	6
Cohort 2	6	1000	6
Phase II (expansion)	50	850	6

### Patients

Eligibility for the dose escalation cohort included patients with a histologically or cytologically confirmed malignant solid tumor that was refractory to all standard therapies. Eligibility for the expansion cohort included patients with metastatic CRC who had progressed on, were intolerant of, or were inappropriate for all standard therapies. Subjects must have been treated with a fluoropyrimidine (e.g., 5-fluorouracil or capecitabine), oxaliplatin, irinotecan and bevacizumab or have contraindication to such treatment. Prior epidermal growth factor receptor (EGFR)-targeting agent (cetuximab or panitumumab) was required for subjects with *RAS* wildtype tumors. Patients in the dose escalation cohort were not required to have measurable disease by RECIST version 1.1. Patients in the expansion cohort were required to have measurable disease by RECIST version 1.1. Inclusion criteria for all subjects in the dose escalation and the expansion cohorts included Karnofsky performance status (KPS) equal to or greater than 70%, life expectancy of at least 3 months, and adequate organ and marrow function.

Exclusion criteria for all subjects in the dose escalation and expansion cohorts included systolic blood pressure greater than 150 mmHg and/or diastolic blood pressure greater than 90 mmHg, history of arterial thromboembolic events or symptomatic pulmonary embolism within 6 months of study enrollment, anti-coagulation with warfarin, history of fistula, history of gastrointestinal perforation, and history of any major bleeding within 6 months of enrollment. Prior treatment with ziv-aflibercept was permitted.

### Safety and DLT assessment

The National Cancer Institute Common Toxicity Criteria version 4.0 (NCI-CTC; version 4.0) was used to assess adverse events (AEs). Enrolled patients were considered evaluable for toxicity if they received any treatment. Patients in the dose escalation cohort were evaluable for DLT if they completed cycle one or experienced a DLT in cycle one. Patients not evaluable for DLT were replaced. The following treatment related adverse events (TRAEs) were considered DLT in cycle 1: any grade 4 neutropenia, thrombocytopenia, or anemia or grade 3 neutropenia or thrombocytopenia lasting more than 7 days; any grade 3 thrombocytopenia associated with bleeding; neutropenic fever; nausea, vomiting or diarrhea grade ≥ 3 and lasting ≥4 days despite supportive measures; grade ≥ 3 bilirubin, ALT or AST elevation > 7 days; other non-hematologic toxicity grade ≥ 3 excluding alopecia, anorexia, fatigue, hypertension, isolated lab abnormalities (not clinically significant) and rare, idiosyncratic reactions to any of the study drugs; inability to receive at least 80% of scheduled doses of each study drug due to treatment-related toxicity; any treatment-related death or treatment-related hospitalization. Anorexia, fatigue, and hypertension were considered dose-limiting if they were unmanageable or were grade 4 in severity.

### Clinical and radiographic assessment

All patients received a clinical assessment at baseline and then every 3 weeks before treatment with ziv-aflibercept. These assessments included vital signs, performance status, and routine laboratory studies. Urinalysis or urine protein to creatinine ratio (UPC) was obtained every 3 weeks (each cycle). In the dose escalation cohort, physical examination and clinical assessment occurred weekly during cycle one.

Radiographic disease assessments were performed using either contrast-enhanced computed tomography (CT) or magnetic resonance imaging (MRI) of the chest, abdomen, and pelvis. These radiographic assessments were completed within 4 weeks prior to the start of therapy and repeated every 3 cycles (9 weeks). Tumors were evaluated using RECIST version 1.1.

### Statistical analysis

The primary study objective of the phase Ib dose escalation cohort was to determine the MTD and RPTD of capecitabine in combination with ziv-aflibercept and to describe any non-dose limiting and dose limiting toxicities. The primary objective of the phase II expansion cohort was to estimate clinical activity. The primary endpoint was 2-month PFS. Secondary endpoints included tumor response, PFS, and OS. Based on historical results the null hypothesis was a 2-month PFS of 48% for capecitabine alone [13] tested against the alternative of a 2-month PFS of 65%. Two-month PFS and its 95% CI were estimated as a binomial proportion. The criterion for success was an observed proportion of at least 30 of 50 patients progression free at 2 months (α = 0.06; β = 0.19). Median PFS and OS were estimated by the Kaplan-Meier method. For tumor response, the frequency of best response (complete response + partial response) over the treatment period was tabulated and its 95% CI was computed.

### Tumor samples

Baseline, formalin-fixed, paraffin-embedded (FFPE) tumor specimens were used for gene expression analyses. Tissue samples of sufficient size for RNA extraction were chosen based on review of pathology reports. Tumor-containing regions were manually macrodissected using a hematoxylin and eosin (H&E) stained slide as a guide.

### RNA extraction and reverse-transcription

Total RNA was extracted from 5 × 5 μm slides using the Maxwell RSC FFPE RNA Kit (Promega, Madison, WI) according to the manufacturer’s instructions. Reverse-transcription of 200 ng of RNA was performed using the High Capacity cDNA Reverse Transcription kit (Applied Biosystems, Foster City, CA) according to the manufacturer’s instructions.

### Real-time PCR

Of the 63 patients that accrued to the study, a total of 31 tumor samples yielded sufficient RNA for reverse-transcription. Expression levels of VEGF-A, VEGF-C, VEGF-D, PlGF, NRP1, and NRP2 were quantified using preformulated TaqMan real-time PCR assays and TaqMan Gene Expression Master Mix (Applied Biosystems, Foster City, CA) in 10 μl reactions according to the manufacturer’s instructions. All assays were run in duplicate. If the standard deviation (SD) in cycle thresholds (CT) between replicate wells was > 0.5 then the reaction was rerun. If the rerun data improved replicate consistency (SD < 0.5), then the rerun data was used. Gene expressions levels were normalized to β-actin using the ΔCT method. Gene-specific assays are listed in Additional file 1: Table S1.

## Results

### Patient characteristics

Subject accrual occurred between September 2012 and October 2015. Sixty-three patients received at least one dose of capecitabine and ziv-aflibercept and were considered evaluable for toxicity. Patient demographics are summarized in Table 2. The median age in this study was 57.2 years (range 35.8–80.7 years); 59% of patients were male. Among 13 patients enrolled and treated in the dose escalation cohort, seven patients had received 3 or more prior lines of treatment. Various tumor types were included in the dose escalation cohort, including colon cancer (5 patients), breast cancer (4 patients), gastric and esophageal cancers (3 patients), and other GI malignancies (1 patient). Fifty patients with metastatic colon and rectal cancer were enrolled and treated in the expansion cohort. Twelve of these patients (24%) received two or fewer prior treatments for metastatic disease. Seventeen patients (34%) received 3 prior treatments, and twelve (24%) received four prior treatments for metastatic disease. Nine patients (18%) received five or more prior treatments. Within the expansion cohort, 46 patients (92%) had received prior 5-FU, twenty-six patients (52%) had received prior capecitabine, and twenty-two patients (44%) had received both capecitabine and 5-FU. Among the patients who received capecitabine prior to enrollment, 81% had experienced disease progression. Among the patients who received 5-FU prior to enrollment, 83% had experienced disease progression. Forty-eight patients (96%) had experienced progression on either 5-FU or capecitabine prior to enrollment. All enrolled patients in the expansion cohort had received prior anti-VEGF therapy (bevacizumab). Within the expansion cohort, forty-five patients (90%) had experienced disease progression on bevacizumab prior to enrollment. One patient had received ziv-aflibercept prior to enrollment (in combination with FOLFIRI), and this patient experienced disease progression.

**Table 2:** Baseline demographics and patient characteristics overall and by cohort

Characteristic	Dose escalation cohort (*n* = 13)	Expansion cohort (*n* = 50)	Overall (*n* = 63)
Age, year			
Mean	58.1	57.7	57.8
Median	55.1	57.2	57.2
Range	40.7–80.7	35.8–75.5	35.8–80.7
Gender, n (%)			
Male	7 (54)	30 (60)	37 (59)
Female	6 (46)	20 (40)	26 (41)
Race			
White	11 (85)	29 (58)	40 (63)
Black	2 (15)	14 (28)	16 (25)
Other / unknown	0	7 (14)	7 (11)
Performance status, KPS (%)			
100	1 (8)	5 (10)	6 (9)
90	7 (54)	18 (36)	25 (40)
80	3 (23)	24 (48)	27 (43)
70	2 (15)	3 (6)	5 (8)
# of prior treatments (%)			
1	1 (8)	2 (4)	3 (5)
2	5 (38)	10 (20)	15 (24)
3	6 (46)	17 (34)	23 (31)
4	0	12 (24)	12 (19)
≥ 5	1 (8)	9 (18)	10 (16)
Tumor Type, n (%)			
Breast	4 (31)	0	4 (6)
Colon	5 (38)	41 (82)	46 (73)
Esophagus	1 (8)	0	1 (2)
Gastric	2 (15)	0	2 (3)
GI	1 (8)	0	1 (2)
Rectum	0	9 (18)	9 (14)

### Dose escalation and RP2D determination

Thirteen patients were enrolled and treated in the Dose Escalation Cohort. In Dose Level 1 (capecitabine 850 mg/m^2^ p.o. bid, days 1–14; ziv-aflibercept 6 mg/kg I.V. day 1 of each 21-day cycle), one subject experienced a DLT event (Grade 3 colonic perforation). One patient in this cohort was not evaluable for DLT assessment due to disease-related intercurrent illness. Capecitabine was then increased to 1000 mg/m^2^, and two of six patients experienced DLT events. These DLT events included grade 2 intolerable fatigue (*n* = 1) and grade 3 oral mucositis (n = 1). Based on one DLT among six evaluable patients, Dose Level 1 was established as the MTD and RPTD.

### Efficacy

**Fig. 1:**
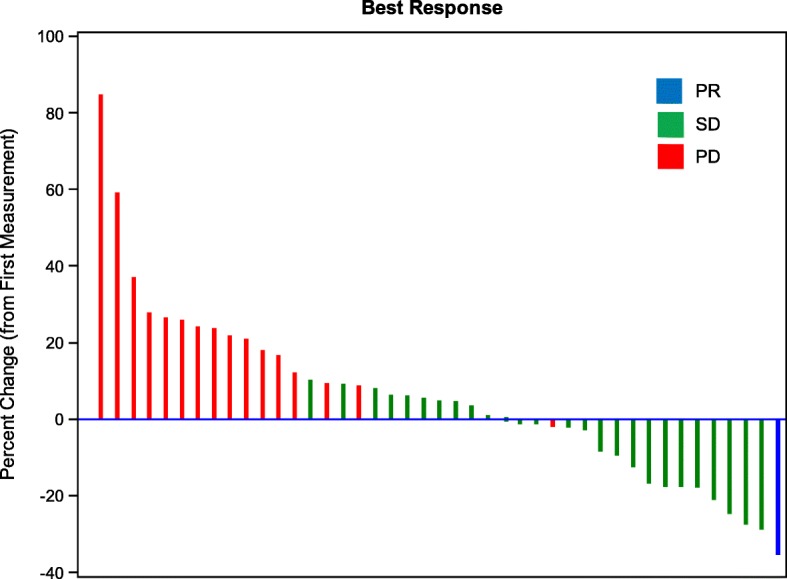
Best response for evaluable subjects (*n* = 45). PR = Partial Response; SD = Stable Disease; PD = Progressive Disease

**Fig. 2:**
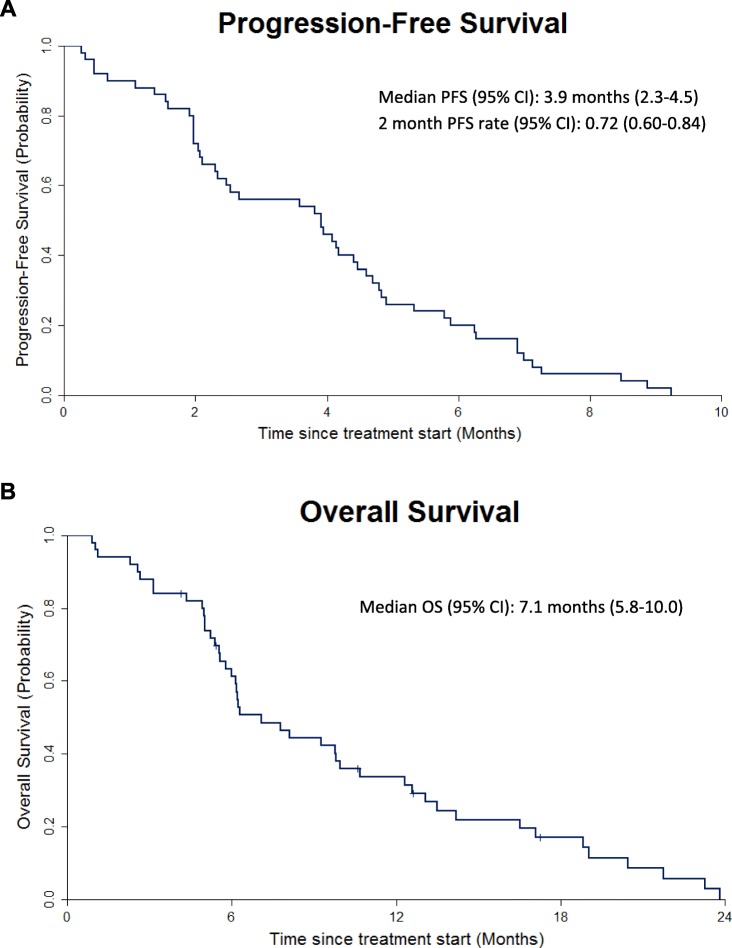
Kaplan-Meier curves for (**a**) progression free survival and (**b**) overall survival

Of the 10 subjects evaluable for tumor response in the dose escalation cohort, seven patients had stable disease (SD) and 3 patients had progressive disease (PD). Within the expansion cohort, one subject experienced a partial response (PR), with an overall response rate (ORR) of 2% (Fig. 1). In the expansion cohort, 72% of patients were progression-free at two months (95% CI, 60–84%), significantly greater than the historical control (48%). Median PFS was 3.9 months (95% CI, 2.3–4.5) (Fig. 2a). Ten patients in the expansion cohort had PFS greater than six months. Overall survival was 7.1 months (95% CI, 5.8–10.0) (Fig. 2b).

### Safety

Table 3 summarizes treatment related adverse events (TRAEs). Grade 1 AEs were not recorded. Of all the patients treated, 52 patients (83%) experienced at least one TRAE. The most common TRAEs of any grade were palmar-plantar erythrodysesthesia syndrome (PPE) (41%), hypertension (33%), oral mucositis (19%), fatigue (16%), and proteinuria (14%). Most AEs were moderate (grade 2) and resolved with supportive clinical care and protocol-specified treatment suspension and dose reductions. 32 patients (51%) experienced a grade 3 or greater TRAE. The most common grade 3 or 4 TRAEs were hypertension (22%), PPE (6%), and mucositis (5%). One patient experienced a Grade 4 intracranial hemorrhage probably related to study treatment (ziv-aflibercept). There was one death from respiratory failure (Death NOS), thought to be related to progressive disease, although a contribution from ziv-aflibercept cannot be excluded.

**Table 3:** Summary of treatment-related adverse events overall and by dose level

AE type reported	850 mg/m2		1000 mg/m2		Total	
	*N* = 57, n(%)		*N* = 6, n(%)		*N* = 63, *n*(%)	
	All events	Grade ≥ 3	All events	Grade ≥ 3	All events	Grade ≥ 3
Patients with AEs	46 (81)	30 (53)	6 (100)	2 (33)	52 (83)	32 (51)
AE by term						
Palmar-plantar erythrodysesthesia syndrome	23 (40)	3 (5)	3 (50)	1 (17)	26 (41)	4 (6)
Hypertension	20 (35)	14 (25)	1 (17)	0 (0)	21 (33)	14 (22)
Oral Mucositis	8 (14)	2 (4)	4 (67)	1 (17)	12 (19)	3 (5)
Fatigue	9 (16)	0 (0)	1 (17)	0 (0)	10 (16)	0 (0)
Proteinuria	9 (16)	2 (4)	NA	NA	9 (14)	2 (3)
Anorexia	3 (5)	1 (2)	2 (33)	1 (17)	5 (8)	2 (3)
Nausea	3 (5)	2 (4)	NA	NA	3 (5)	2 (3)
Platelet count decreased	4 (7)	2 (4)	NA	NA	4 (6)	2 (3)
Dehydration	2 (4)	2 (4)	NA	NA	2 (3)	2 (3)
Intracranial Hemorrhage	1 (2)	1 (2)	NA	NA	1 (2)	1 (2)
Death NOS	1 (2)	1 (2)	NA	NA	1 (2)	1 (2)

### Correlative studies

To determine whether the expression of angiogenic ligands correlated with clinical benefit, real-time PCR was performed to evaluate baseline levels of VEGF-A, VEGF-C, VEGF-D, PlGF, NRP1, and NRP2 expression. VEGF-D was excluded from this analysis as it could not be reliably detected in these samples. No associations were observed between gene expression levels (ΔCt) and survival outcomes (PFS and OS). Furthermore, when patients with PR/SD were compared to patients with PD, gene expression did not significantly differ between the responder and non-responder groups (Additional file 1: Table S2).

## Discussion

Angiogenesis plays a vital role in mediating embryonic development, wound healing, and vascular permeability, but can also be co-opted by tumors to promote growth and metastasis [19, 20]. Ziv-aflibercept—which binds ligands for VEGFR1 and VEGFR2 and prevents receptor activation—is a potent angiogenesis inhibitor. Although the survival benefit of ziv-aflibercept is well established for the second line treatment of metastatic CRC, its activity is not well established in patients treated in the third line setting and beyond.

In this phase Ib/II study, we established the MTD/RPTD of capecitabine and ziv-aflibercept in patients with refractory advanced solid tumors. The MTD/RPTD of capecitabine in this study (850 mg/m^2^, p.o. bid, days 1–14 of each 21 day cycle) is lower than the dosage listed in the capecitabine prescribing information (1250 mg/m^2^, p.o. bid, days 1–14 of each 21 day cycle) [21]. It is possible that this lower RPTD of capecitabine reflects the heavily pretreated patient population. Additionally, regional variation in capecitabine tolerability may have impacted the RPTD [22]. Alternatively, ziv-aflibercept may have potentiated the effects of chemotherapy, thereby reducing the tolerability of capecitabine. The phase III VELOUR study noted greater rates of chemotherapy-related toxicity in patients receiving ziv-aflibercept, including diarrhea, stomatitis, PPE, and neutropenia [6]. However, even with the lower RPTD of capecitabine, this study suggests that efficacy was not compromised. As shown in a prior study, capecitabine dosage and schedule may impact toxicity, but response rate—a surrogate for clinical benefit— remains similar [23].

In the expansion cohort, the combination of capecitabine and ziv-aflibercept had acceptable tolerability at the RPTD. Treatment related grade 4/5 AEs were rare (3%). The most common TRAEs—PPE, hypertension, and oral mucositis—were anticipated, and were in most cases successfully managed with treatment suspension and dose modifications. Only 9% of patients (5/57) treated at the RPTD discontinued treatment due to toxicity. Given the favorable tolerability of capecitabine and ziv-aflibercept compared to available alternatives, this may be a regimen well suited to patients with advanced age or comorbidities.

In this study, the combination of capecitabine and ziv-aflibercept met its primary goal to demonstrate a 2-month PFS statistically greater than 48%. The median PFS observed in this study— 3.9 months (95% CI, 2.3–4.6)—compares favorably to historical controls from standard of care therapies, including TAS-102 (median PFS = 1.9 months), regorafenib (median PFS = 1.9 months), and capecitabine monotherapy (median time to progression = 2 months) [2, 3, 13]. Additionally, the median OS of 7.1 months (95% CI, 5.8–10.0) observed in this study is similar to historical controls from TAS-102, regorafenib, and capecitabine monotherapy [2, 3, 13]. Although only one patient achieved a confirmed RECIST partial response, the ORR of TAS-102 and regorafenib are also less than 2% [2, 3]. The primary clinical benefit of capecitabine and ziv-aflibercept observed in this study was disease stabilization, which occurred in the majority (60%) of 45 evaluable patients with metastatic CRC. Disease stabilization occurred despite prior progression on 5FU or Xeloda in 96% of enrolled patients, and prior progression on bevacizumab in 90% of enrolled patients. Given the absence of a control population and limited number of participating sites, these results will need confirmation in a larger randomized study. Recent evidence further supports the clinical benefit of oral cytotoxic chemotherapy combined with an anti-VEGF monoclonal antibody. A randomized study in patients with chemotherapy refractory metastatic CRC demonstrated improved PFS for TAS-102 and bevacizumab compared with TAS-102 alone (Median PFS, 5.9 months versus 2.9 months; *P* < 0.05) [24]. These results support the therapeutic approach, and offer a potential cytotoxic chemotherapy partner (TAS-102) for future investigation.

In this study, some patients experienced exceptional clinical benefit. This benefit occurred despite prior treatment with anti-VEGF targeting therapies. In the dose escalation cohort, a patient with metastatic breast cancer who had received 12 lines of prior therapy—including capecitabine/ bevacizumab—experienced significant reduction in symptomatic ascites, and stable disease for over 6 months. A second subject with treatment refractory breast cancer experienced stable disease for over 8 months. Within the expansion cohort, two subjects experienced stable disease lasting longer than 6 months. Correlative analyses did not identify an association between the expression of angiogenesis-associated genes and patient outcomes. Assays of additional genes may yet identify actionable predictive biomarkers. This analysis—with its limited number of samples and genes assayed—highlights the challenges of identifying predictors of benefit from anti-VEGF therapies.

## Conclusion

The combination of capecitabine and ziv-aflibercept was tolerable and met its pre-specified target for clinical efficacy. Additional study in a prospective randomized clinical trial is warranted. Further study is needed to better define the role of biomarkers in predicting sensitivity and resistance to anti-VEGF therapy.

## Additional file

**Additional file 1: Table S1.** Taqman Gene Expression Primers and **Table S2**. Association of angiogenic ligand expression with clinical outcomes.

## Data Availability

The data that support the findings of this study are available from the corresponding author upon reasonable request.

## Abbreviations

5-FU: 5-Fluorouracil
AE: Adverse event
ALT: Alanine aminotransferase
AST: Aspartate aminotransferase
CBC: Complete blood count
CI: Confidence interval
CNS: Central nervous system
CRC: Colorectal cancer
CT: Computed tomography
CT: Cycle thresholds
DLT: Dose limiting toxicity
DNA: Deoxyribonucleic acid
EGFR: Epidermal growth factor receptor
EMA: European Medicines Agency
FDA: Food and Drug Administration
FFPE: Formalin-fixed, paraffin-embedded
FLK1: Fetal Liver Kinase 1
FLT1: fms related tyrosine kinase 1
FOLFIRI: 5-Fluorouracil, leucovorin, and irinotecan
GI: Gastrointestinal
H&E: Hematoxylin and eosin
HR: Hazard ratio
I.V.: Intravenous
KDR: Kinase insert domain receptor
KPS: Karnofsky Performance Status
mmHg: Millimeters of mercury
MRI: Magnetic resonance imaging
MTD: Maximum tolerated dose
NCI-CTC: National Cancer Institute Common Toxicity Criteria
NRP1: Neuropilin 1
NRP2: Neuropilin 2
ORR: Objective response rate
OS: Overall survival
P.O.: Per os (by mouth)
PCR: Polymerase chain reaction
PD: Progressive disease
PFS: Progression-free survival
PlGF: Placental growth factor
PPE- palmar: Plantar erythrodysesthesia
PR: Partial response
*RAS* wild-type: Negative for mutations in *KRAS* or *NRAS* exons 2, 3, or 4
RECIST: Response evaluation criteria in solid tumors
RNA: Ribonucleic acid
RPTD: Recommended phase II dose (RPTD)
SD: Stable disease
SD: Standard deviation
TRAE: Treatment related adverse events
UPC: Urine protein to creatinine ratio
VEGF-A: Vascular endothelial growth factor-A
VEGF-C: Vascular endothelial growth factor-C
VEGFR1: Vascular endothelial growth factor receptor 1
VEGFR2: Vascular endothelial growth factor receptor 2
β-HCG: Beta-human chorionic gonadotropin
