# Supplementary material for: A phase Ib study of capecitabine and ziv-aflibercept followed by a phase II single-arm expansion cohort in chemotherapy refractory metastatic colorectal cancer
Source: BMC Cancer. 2019 Nov 1;19:1032. doi: 10.1186/s12885-019-6234-8 (PMC6824108; doi:10.1186/s12885-019-6234-8)
Supplement: Supplementary file 1 — Additional file 1: Table S1. Taqman Gene Expression Primers and Table S2. Association of angiogenic ligand expression with clinical outcomes. [file 12885_2019_6234_MOESM1_ESM.docx]

**Additional file 1**

**Table S1: Taqman Gene Expression Primers**

| **Gene** | **Assay** |
| --- | --- |
| β-actin | Hs00357333_g1 |
| NRP1 | Hs01546494_m1 |
| NRP2 | Hs01033058_m1 |
| PlGF | Hs00182176_m1 |
| VEGF-A | Hs00900055_m1 |
| VEGF-C | Hs01099203_m1 |
| VEGF-D | Hs01128657_m1 |

**Table S2: Association of angiogenic ligand expression with clinical outcomes**

|  | PFS | | OS | |
| --- | --- | --- | --- | --- |
| Gene | HR | p-value | HR | p-value |
| NRP1 | 0.91 (0.73 – 1.13) | 0.39 | 0.93 (0.72 – 1.19) | 0.34 |
| NRP2 | 0.96 (0.71 – 1.29) | 0.76 | 0.996 (0.71 – 1.40) | 0.98 |
| PlGF | 0.78 (0.51 – 1.19) | 0.25 | 0.78 (0.48 – 1.27) | 0.32 |
| VEGF-A | 0.95 (0.61 – 1.48) | 0.82 | 0.85 (0.51 – 1.41) | 0.52 |
| VEGF-C | 1.07 (0.81 – 1.42) | 0.62 | 0.98 (0.71 – 1.35) | 0.91 |

| PR/SD vs PD | |
| --- | --- |
| Gene | p-value |
| NRP1 | 0.98 |
| NRP2 | 1.00 |
| PlGF | 0.90 |
| VEGF-A | 0.82 |
| VEGF-C | 0.57 |
